# Towards Wearable Health Monitoring Devices

**DOI:** 10.3390/bios12050322

**Published:** 2022-05-11

**Authors:** Vladimir A. Pozdin, James Dieffenderfer

**Affiliations:** 1 Department of Electrical and Computer Engineering, Florida International University, Miami, FL 33174, USA; 2 Department of Mechanical and Materials Engineering, Florida International University, Miami, FL 33174, USA; 3 Department of Electrical and Computer Engineering, North Carolina State University, Raleigh, NC 27606, USA; jpdieffe@ncsu.edu

Humans have searched far beyond our planet to understand the fundamental principles and mechanisms of life. Although this search has extended outwards past our Earth, the recent advances in micro and soft electronics have shifted the focus back inwards—understanding how our bodies function and how to improve and prolong their lifespan. Research has even ventured inside the human body to explore the fundamental principles and building blocks of human thought with spatial and temporal recordings of neuronal activity. Biomarker and performance monitoring are seen as key elements to maintaining our health, identifying detrimental activities, and reinforcing healthy habits. The recent global pandemic has underscored the importance of health and environmental monitoring for complete and up-to-date information on our health and to guide us to a higher quality of life and greater worldly advances.

Advances in microelectronics, such as the development of flexible and stretchable circuits boards, have led to a boom in wearable sensors; the watershed moment of having low-cost health monitoring and diagnostic devices is upon us. Monetization of personal data by technology giants has demonstrated that our uniqueness can be analyzed, quantified, and predicted. These advances have fueled research into quantification and predication of health, particularly in the area of neurological diseases and disorders. As most neurological diseases do not manifest until late stages of the disease, current focus is on early detection and development of cures for pre-terminal patients. Advances in computational algorithms on established datasets show great promise in achieving these goals; however, the pandemic has shown a great disparity in existing datasets, as common pulse oximeters fail to provide accurate results for individuals with non-white skin pigmentation [1]. These failures expose the need for large universal datasets, which could lead to advances for the entire human race. In addition, early detection data are needed for patients outside of a hospital setting to identify early stage disease biomarkers. To achieve this level of monitoring, the sensors need to evolve from benchtop financial behemoths to low-cost portable sensors, which can seamlessly be part of our daily life. As technology advances towards portable sensing, one of the challenges is the integration of all subsystems into an unobtrusive device for continuous data collections (Figure 1). Significant breakthroughs are possible through the deployment of sensors to the wider public for unobtrusive long-term monitoring, as it is not feasible to subject participants to life threatening conditions to advance early detection.

Unobtrusive long-term monitoring devices require focus on materials research, circuit design, fabrication, user interaction, and data processing. Conformable circuit design is needed to attach devices to the skin without strong adhesives and achieve low noise levels. Fabric integration is a viable route for a wide range of sensors, including environmental monitoring, however significant durability improvements of textile devices are needed for deployment. Wearable devices require noise and motion resilient circuitry and algorithms, as well as multimodal sensing to provide user’s context for the measurements. One of the biggest challenges of wearable sensors is the power source to achieve reusable long-term operation, as device safety requires encapsulation, and ideal source is self-contained, non-hazardous, and durable. Importantly, device communication and user integration are vital to achieve user adherence and long-term data collection. Due to vast design criteria of wearable sensors, numerous sensors demonstrated for wearable detection of biomarkers utilized benchtop hardware, lacked wireless communication, relied on proprietary software for data analysis, or lacked a power module. Extensive collaboration across the scientific community is needed to advance the research towards truly wearable and imperceptible sensors. 

In this Special Issue, we strive to highlight the development of wearable sensors across the areas of sensor design, flexible circuitry, and computation to motivate interdisciplinary research to realize complete wearable systems. Complete wearable systems with supporting computation and interfaces allow for the engagement of medical professionals, allowing for inward look within ourselves as humans, to seek out the answers to increasing our quality of life and reversing the burden of incurable diseases.

## Figures and Tables

**Figure 1:**
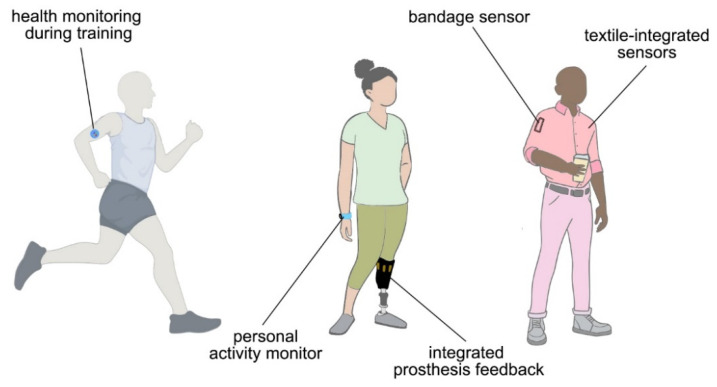
Wearable sensor past, current, and future. Early sensors focused on monitoring training of military personnel and athletes. Current personal electronics boom integrates activity and health monitoring into existing electronics (smart phones, watches, prosthetics). Future wearable devices aim to seamlessly integrate into our daily life for continuous feedback on our health. (This image was created using BioRender, Office, and Adobe products.).
